# The value of walking: a systematic review on mobility and healthcare costs

**DOI:** 10.1186/s11556-022-00310-3

**Published:** 2022-12-29

**Authors:** Martin Wohlrab, Jochen Klenk, Laura Delgado-Ortiz, Michael Chambers, Lynn Rochester, Matthias Zuchowski, Matthias Schwab, Clemens Becker, Simon U. Jaeger

**Affiliations:** 1 Dr. Margarete Fischer-Bosch-Institute of Clinical Pharmacology, Auerbachstrasse 112, 70376 Stuttgart, Germany; 2 University of Tuebingen, Tuebingen, Germany; 3 Robert-Bosch Gesellschaft Für Medizinische Forschung, Stuttgart, Germany; 4 Institute of Epidemiology and Medical Biometry, Ulm University, 89081 Ulm, Germany; 5 IB University of Health and Social Sciences, Study Center Stuttgart, 70178 Stuttgart, Germany; 6 ISGlobal, Barcelona, Spain; 7 Universitat Pompeu Fabra, Barcelona, Spain; 8 CIBER Epidemiología Y Salud Pública, Barcelona, Spain; 9 MC Healthcare Evaluation, London, UK; 10 Translational and Clinical Research Institute, Newcastle Unviersity, Newcastle, UK; 11 Robert-Bosch-Hospital, Stuttgart, Germany; 12 Depts. of Clinical Pharmacology, and of Biochemistry and Pharmacy, University of Tuebingen, Tuebingen, Germany; 13 Unit Digital Geriatric Medicine, University Clinic Heidelberg, Heidelberg, Germany; 14 Department of Clinical Pharmacology, University Hospital, University of Tuebingen, Tuebingen, Germany

**Keywords:** Walking, Mobility, Health care costs, Systematic review

## Abstract

**Background:**

The ability to walk is an important indicator of general health and mobility deficits have wide-ranging economic implications. We undertook a systematic review to elucidate the impact of walking parameters on health care costs.

**Methods:**

Publications reporting on associations between health care costs and walking parameters were identified by a systematic literature search in MEDLINE, Embase, and manual reference screening, following the PRISMA reporting guidelines. First, titles and abstracts were screened by two independent reviewers followed by a review of the full articles if they met the inclusion criteria. Costs were converted to US-Dollars with inflation adjustment for 2021. A narrative synthesis was performed.

**Results:**

Ten studies conducted between 2001 and 2021 fulfilled the inclusion criteria. Assessment of walking ability was carried out via patient reported outcomes, performance tests, or using wearable digital devices. Walking more than one hour per day, a faster walking speed and the ability to walk without impairments are associated with significant lower health care costs. A higher number of steps per day is associated with significant lower costs in two simulation studies, while in the study using a digital device, taking more than 10,000 steps per day is not significantly associated with lower direct costs. The heterogeneity of mobility assessments and of economic analyses both precluded a quantitative synthesis.

**Conclusion:**

Cross-sectional and observational studies from this systematic review suggest a significant association of better walking performance with lower health care costs. Future health economic research and health technology assessments should use quantifiable mobility outcomes when evaluating new drugs or non-pharmacological interventions.

## Introduction

Walking ability has emerged as important indicator of general health and has even been proposed to be a ‘vital sign’ [1, 2]. Significant correlations between walking parameters (e. g. gait speed or sedentary time) and health outcomes such as mortality, morbidity, and quality of life have been established in recent years [3–5]. For example, taking more steps per day is associated with progressively lower all-cause mortality for young-middle age adults as well as for older adults [6]. In hip fracture patients, the ability to walk is a strong indicator of long-term survivorship [7], and Parkinson's disease patients with disturbed movement and physical impairments more likely experience falls [8]. Walking related adverse events (e.g. falls, admissions to care homes and hospitalisation), are recognized as crucial drivers of the costs of patient management [9, 10].

Walking ability covers a meaningful aspect of health and reflects how a patient functions in daily life. As such, it represents an important outcome for studies on health-prevention and on effects of rehabilitation or surgery. Nonetheless, in the area of pivotal clinical trials, which are essential stages in drug development and marketing authorisation of new medicines, we have shown that assessments of walking parameters are not routinely included [11]. This is regrettable, since improvement in walking performance, in addition to disease-specific clinical efficacy and safety, represents a patient-relevant benefit. A positive effect of a new drug on the patients’ walking performance could also strengthen the manufacturer position in health technology assessments (HTA) or reimbursement negotiations. The constrained budgets of most national health systems mandate a careful allocation of health resources, and improvement or worsening in walking ability as a consequence of treatment could be an interesting additional variable in economic models for cost-effectiveness and cost utility analyses. Yet, a better understanding is still needed of how walking ability and costs are linked at the patient level and which parameters are the best estimators for costs.

At present, clinical trials assess walking most often as a part of generic quality of life questionnaire scores (e. g. EQ-5D, SF-36) which include some components/questions concerning mobility [11]. Tangible information on actual walking status is difficult to derive from the summary scores. Conventional performance outcomes measuring walking-related mobility (e. g. the 6-min walking distance) may suffer from other limitations, e.g. a moderate external validity [12]. Developments in digital technology have opened up the way for comprehensive real-world measurements of mobility including walking volume, pace, variability, asymmetry and phases [13]. Although the first steps have already been taken to establish walking performance as endpoints in clinical trials (for example using stride velocity as a secondary endpoint in Duchenne Muscular Dystrophy [14]), validation of these novel digital mobility outcomes (DMOs) is an important prerequisite. Mobilise-D (https://www.mobilise-d.eu) [15]*,* an EU-funded collaborative project of academia and industry, is dedicated to promote the acceptance of DMOs by regulatory authorities as endpoints in clinical trials, and by implication its use in labelling and marketing of new medicines.

The role of assessing walking ability for the analysis or prediction of health care expenditure is currently not well understood, as is its potential contribution to economic evaluations of new interventions. The present study aims to identify and synthesize the available primary literature investigating the specific association of walking parameters (conventional and digital) and health care costs.

## Methods

This systematic review was conducted according to the Centre for Reviews and Dissemination’s Guidance for undertaking reviews in healthcare, with adherence to the Preferred Reporting Items for Systematic Reviews and Meta-Analyses (PRISMA) reporting guidelines [16]. The review has been registered in PROSPERO, the international database of prospectively registered systematic reviews (CRD42021261443).

### Eligibility criteria

**Fig. 1:**
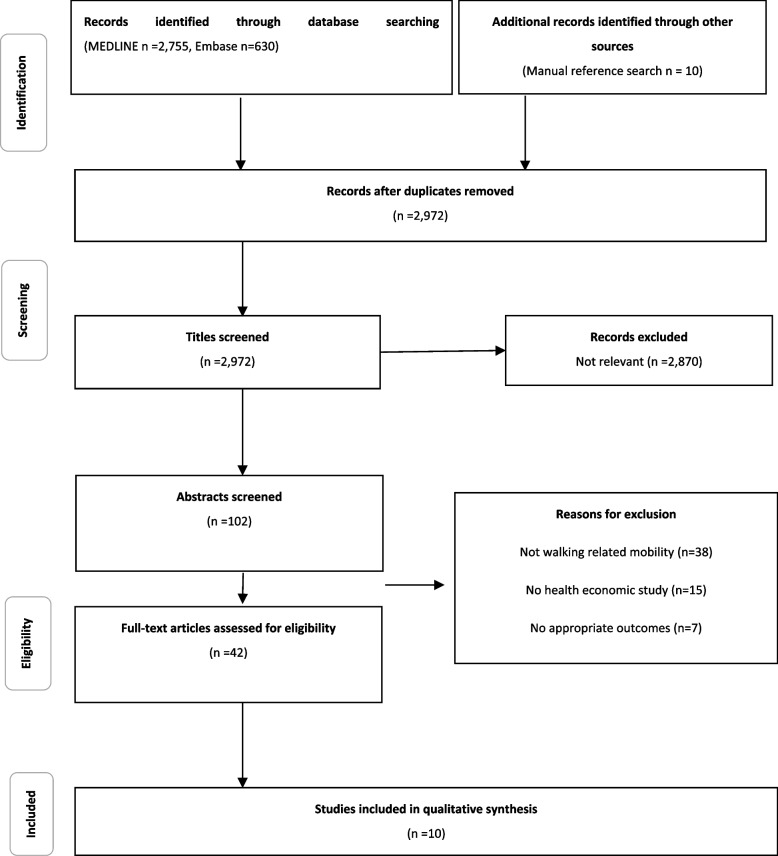
Flow chart of search results (adapted from PRISMA Flow Diagram)

Eligibility criteria were defined using the PICO scheme (see appendix). The population was not restricted to specific indications, ages, gender or geographic location. This review only includes studies assessing the economic consequences of mobility in terms of health care costs. We specifically focused on walking-related mobility parameters including gait speed, walking speed, or the number of steps, among others. Vector magnitude units per minute (VMU) or energy expenditure which are not necessarily derived from walking and questionnaires evaluating additional activity parameters at the same time were excluded. Studies that examined relationships between mobility and quality of life or further clinical factors (e. g. falls or mortality, which could have implications on health care costs), but did not assess economic consequences directly, were excluded. A PRISMA flow chart shows the study selection process, also giving reasons for exclusion (Fig. 1).

### Information sources and search strategy

MEDLINE and EMBASE were searched for relevant primary research publications published from database inception until November 2022. The search was supplemented by manual reference screening and cross-referencing. The search strategy comprised two main constructs that refer to walking-related mobility outcomes and the economic implications. The main keywords “mobility”, “walking speed”, and “cost” were searched without restriction, thus not limiting the search strategy to the type of (cost) analysis. Additional search terms included in the search strategy were confined to the title. The search was restricted to studies of human subjects, written in the English language. Editorials, letters, historical articles, abstracts, and reviews were excluded. The full search strategy is presented in the Appendix.

### Study selection

Two reviewers (MW, SJ) independently screened titles of all identified studies. If either reviewer considered a study to meet the inclusion criteria, its abstract was then screened independently. Upon agreement on inclusion, full texts were retrieved and reviewed independently. Disagreements were resolved in discussions with a third reviewer (MZ). Data merging, deduplication and screening was performed with the open source R package revtools [17].

### Data extraction

Data were extracted from selected studies for the following study characteristics: author and year, title of publication, country of investigation, study perspective and comparators, study population and disease area, study design, database and sample size, type of medication, and cost results distributed across sectors (e. g. inpatient, outpatient, emergency, prescriptions, total costs) where reported. To facilitate comparison across studies, all costs from different country sources were inflated to corresponding values for the year 2021, using local inflation rates. These were converted to US dollars (USD) values based on 2021 end of year exchange rates published by the US Federal Reserve [18].

### Quality of reporting assessment

We assessed the reporting quality of studies following on the basis of the Consolidated Health Economic Evaluation Reporting Standards (CHEERS) guideline [19] Given that studies are very heterogeneous and do not completely meet type of studies for which CHEERS was initially implemented (cost evaluations of health interventions), individual items from the checklist were adopted based on the notation by Rothfuss et al. [20] (Table 1).

**Table 1:** Assessment of quality

Study	Background and aims	Perspective	Population	Time horizon	Comparison	Economic outcomes	Discounting	Incremental costs	Sensitivity analysis	Findings and limitations	Funding	Conflict of interest
**Perkins et al. 2001** [21]	+	[ +]	+	+	+	+	N/A	+	0	+	+	0
**Tsuji et al. 2003** [22]	+	[ +]	+	+	+	+	+	+	0	+	0	0
**Purser et al. 2005** [23]	+	[ +]	+	+	+	+	N/A	+	+	+	+	0
**Kato et al. 2013** [24]	+	0	+	+	+	[ +]	0	+	[ +]	+	+	+
**Turi et al. 2015** [25]	+	[ +]	+	+	+	[ +]	+	+	0	+	0	0
**Kabiri et al. 2018** [26]	+	[ +]	+	+	+	[ +]	0	+	+	+		+
**Karl et als. 2018** [27]	+	[ +]	+	+	+	+	+	+	0	+	+	+
**Bonnini et al. 2020** [28]	+	[ +]	+	+	+	+	0	+	0	+	+	+
**Okayama et al. 2021** [29]	+	[ +]	+	+	+	+	0	+	0	+	+	+
**Hirai et al. 2021** [30]	+	[ +]	+	+	+	+	0	+	+	+	+	+

Legend: + , present; [ +], partly fulfilled; 0, absent; N/A, non-applicable; notation based on Rothfuss et al

## Results

### Search results

Results are presented as statistically significant differences of costs in individual studies. Due to heterogeneity in types of cost outcomes, settings, and disease areas, it was not appropriate to synthesize the results or conduct a meta-analysis of the economic findings. The initial search strategy yielded 2,771 titles after the elimination of duplicates. For the final qualitative synthesis, ten studies were included. These studies were conducted in Japan (*n* = 4), United States (*n* = 3), Brazil (*n* = 1), Italy (*n* = 1), and Germany (*n* = 1).

### Study characteristics

Studies were conducted between 2001 and 2021. Detailed information on the individual studies is shown in Table 2. Six studies were observational cohort studies [21–23, 28, 29], two were cross-sectional studies [25, 27], one was a decision analysis using a Markov Model [24], and one was a microsimulation [26]. All studies (*n* = 10) examined the economic impact of mobility in middle-aged to aged populations. All studies focused on direct healthcare costs (including total costs but also costs by individual health sector).

**Table 2:** Study characteristics

Publication	Title	Country	Database and sample size	Study design	Study population and setting	Method & Comparison	Outcome measures	Cost data
**Walking Time**								
Perkins et al. 2001 [21]	Assessing the Association of Walking with Health Services Use and Costs among Socioeconomically Disadvantaged Older Adults	USA	*n* = 1,088 Regenstrief Physical activity and Health Survey (RPAHS)	Cohort study	Community dwelling patients > 55 years	Multivariate models assessing the association between walking and health services use and costs (adjusting for sociodemographic characteristics, chronic disease, health status, and previous utilisation)	Health services use, and total costs	Health services use and cost data obtained from the RPAHS for the 12 months following interviews (Primary care, emergency, hospital costs as charges calculated on an annual basis)
Tsuji et al. 2003 [22]	Impact of walking upon medical care expenditure in Japan	Japan	*n* = 27,431 NHI claims history files	Prospective cohort study	Japanese men and women, aged 40–79 years. National Health Insurance (NHI) beneficiaries in rural Japan	Logistic regression model and multivariate models to adjust for the effect of potential socio economic confounders. Persons walked for < 30 min, 30 min – 1 h, > 1 h (per day)	Medical care Expenditure	Insurance claims history; uniform national fee schedule determines prize for each service prospectively collected for 4 years (charges for outpatient and inpatient care)
Hirai et al. 2021 [30]	Physical Activity and Cumulative Long-Term Care Cost among Older Japanese Adults: A Prospective Study in JAGES	Japan	*n* = 34,797 Japan Gerontological Evaluation Study (JAGES)	Prospective cohort study	Community-dwelling people aged 65 years or older, with no physical or cognitive disabilities	Generalized linear model with Tweedie distribution and log-link function, adjusted for socio economic confounders. Comparison of individuals by 3 categories (< 30 min, 30–59 min, 60 min) of time spent walking	Cost of long-term care insurance services	The outcome variable was the cumulative cost of LTCI services during the follow-up period. Documented in the Japan Gerontological Evaluation Study (JAGES)
**Walking in Leisure Time**								
Turi et al. 2015 [25]	Walking and health care expenditures among adult users of the Brazilian public healthcare system: retrospective cross-sectional study	Brazil	*n* = 963 Basic Health Units	Retrospective cross-sectional study	Patients aged ≥ 50 years	Logistic regression model and multivariate models to adjust for the effect of potential socio economic confounders. Walking (never, seldom, sometimes, often, always) during leisure-time and healthcare expenditure in primary care	Total medical expenditures	Expenditure on consultations, laboratory tests and medical consultations transformed into currency by standard table (provided by Brazilian government)
**Walking Speed**								
Purser et al. 2005 [23]	Walking speed predicts health status and hospital costs for frail elderly male veterans	USA	*n* = 1,388 Department of Veterans Affairs (VA) multicenter clinical trial	Cohort study	Medical or surgical patients > 65 years. Geriatric Evaluation and Management (GEM) program	Multivariate models to adjust for the effect of potential socio economic confounders, assessing baseline in gait speed and absolute change over 1 year	Inpatient health services use, and total costs	Data on length of stay, number, and charges of inpatient consultations, rehabilitation and social work visits as available from Veterans Affairs databases
Bonnini et al. 2020 [28]	Improving walking speed reduces hospitalization costs in outpatients with cardiovascular disease. An analysis based on a multistrata non-parametric test	Italy	*n* = 649 Exercise-based secondary prevention program	Prospective cohort study	Patients participating in an exercise-based secondary prevention program (average age 63)	Multi-strata permutation test after propensity score matching. Patients divided at baseline into two groups characterized by low and high WS (based on the average WS maintained during a moderate 1-km treadmill-walking test)	All-cause hospitalization and related costs	Hospitalization related costs (Process of transformation of hospitalization rates to costs is not described.)
Okayama et al. 2021 [29]	Clinical impact of walking capacity on the risk of disability and hospitalizations among elderly patients with advanced lung cancer	Japan	*n* = 60 Shizuoka Cancer Center	Prospective cohort study	Patients aged ≥ 70 years with advanced non-small-cell lung cancer (NSCLC)	Recurrent event analysis comparing (not adjusted for socioeconomic confounders) comparing Mobile group vs less mobile group	Length of hospital stay, inpatient medical costs	Inpatient medical costs. Electronic health records of hospitals, actual revenue that the hospital was paid from the health insurance funds for a given inpatient stay
**Number of Steps**								
Kato et al. 2013 [24]	Effects of walking on medical cost: A quantitative evaluation by simulation focusing on diabetes	Japan	*n* = 1,000 hypothetical subjects	10 year- Markov model	Hypothetical subjects representing middle aged Japanese people	Markov Model (rates of events were determined on papers and statistical data published in Japan) Patients after 10 years with 0 steps, + 3,000 steps, and + 5,000 steps	Total number of events during 10 years, Medical costs during 10 years	Medical costs for diabetes (inpatient and outpatient costs) associated with each health status events estimated from public statistical data in Japan
Karl et al. 2018 [27]	Direct healthcare costs associated with Device-based assessment, and self-reported physical activity: results from a cross-sectional population-based study	Germany	*n* = 477 KORA FF4 study	Retrospective cross-sectional study	Patients aged between 48 and 68 years	Two-part gamma regression model adjusted for potential socioeconomic confounders comparing inactive participants to active subjects (very low moderate-vigorous physical activity (MVPA) vs. very high MVPA)	Total healthcare costs	Direct medical costs (based on physician visits, ambulatory hospital visits, and drugs) calculated using national unit costs, as recommended by the Working Group Methods in Health Economic Evaluation (AG MEG)
Kabiri et al. 2018 [26]	The Long-Term Health and Economic Value of Improved Mobility among Older Adults in the United States	USA	n = 12.6 million Medical Expenditure Panel Survey (MEPS 2012)	Micro Simulation	Patients ≥ 51 years with osteoarthritis	Six-step process to model the effect of improved mobility though improvements in quality of life measures on health economic outcomes. Comparing “status quo” population (pre-treatment mobility levels) with the “mobility improvement” population	Medical expenditures, Nursing home utilisation	THEMIS microsimulation tracked individuals to translate changes in quality of life to health economic outcomes derived from MEPS data base (included costs were not described)

The description of the source of cost data and the calculation of costs varied considerably across studies. A detailed description was absent in one study [28] but it can be assumed that estimates of hospitalization rates were based on a regional Health Service Registry. Outpatient/emergency room visits or inpatient hospital stays were gathered from institutional electronic health records [21, 25, 29], or insurance databases [22, 23]. Karl et al. [27] directly questioned patients. Hirai et al. [30] used data from the Japan Gerontological Evaluation Study (JAGES), which collected information about the costs from the municipalities that also act as insurers. Kato et al. estimated costs from public statistical data in Japan [24] and the microsimulation study by Kabiri et al. used THEMIS (The Health Economic Medical Innovation Simulation) to estimate how mobility improvements affect medical expenditures through monetized quality adjusted life years including data from MEDICARE, and MEDICAID [26].

### Quality of reporting

The assessed quality of reporting is shown in Table 1. All publications outlined the background adequately to understand the research need and the research question. All studies reported cost differences due to a change in walking parameters. Costs that should be included in an analysis depend on the study perspective (refers to the point of view one takes when assessing costs), so failing to state the perspective meant that some of these studies lacked a clear rationale for the types of cost included. Five studies [24, 26, 28, 29] did not adjust costs that occurred at different points in time, and two studies [23, 26] conducted a sensitivity analysis to address a certain variety of their assumptions.

### Reported results

Cost results are shown in Table 3. Perkins A, Tsuji I, Purser JL, Kato M, Turi B, Kabiri M. [21–26, 28, 29] reported that lower levels of walking ability were associated with higher health care expenditure, and one study [27] reported no statistically significant association between mobility and health care costs. Six studies reported additional associations between walking parameters and health care utilisation [21–23, 26, 28, 29].

**Table 3:** Cost results

Publication	Walking outcomes	Utilization results	Direct Costs results (adjusted to 2021 USD)
Perkins et al. 2001 [21]	PRO: Walking Time (average minutes of walking per week by newly developed questionnaire)	Walking 120 or more minutes was associated with a lower risk of emergency room visit (OR = 0.5) and hospital stay (OR = 0.6) in the subsequent year	Total annual costs for those walking 0 min per week (8,123 $) vs. those walking more than 120 min per week (2,844 $) Inpatient costs for those walking 0 min per week (6,018 $) vs. those walking more than 120 min per week (1,814 $) Emergency room for those walking 0 min per week (1,109 $) vs. those walking more than 120 min per week (388 $)
Tsuji et al. 2003 [22]	PRO: Walking Time (self-reported walking duration per day (3 factor levels))	Compared with those walking > = 1 h/day, per capita per month cost for inpatient care in those walking > = 30 min was 16% higher. For outpatient care, both the number of visits and the medical cost also significantly increased with shorter walking time	Medical costs significantly reduced with longer time spent walking. Per capita medical cost was 173 $ per month in those who walked for 30 min/day, 168 $ in those who walked for 30 min–1 h, and 115 $ in those who walked for more than 1 h
Purser et al. 2005 [23]	PerfO: Walking Speed (timed 50 feet walking trial as part of the ‘Reuben’s Physical Performance Test’)	Each 0.10 m/s reduction in baseline walking speed was associated with additional rehabilitation visits (1.4 to 2.5), increased medical-surgical visits (1.9 to 3.7), and longer hospital stays (1.4 to 2.9) Each 0.10 m/s/yr increase in walking speed resulted in fewer hospitalization days (2.3 [1.3 to 3.3])	Each 0.10 m/s reduction in baseline walking speed was associated with higher costs (1,854 $ [1,207 $ to 2,499 $]) Each 0.10 m/s/yr increase in walking speed resulted in 1-year cost reductions of 1,651 $ [–90 $ to 3,394 $]
Kato et al. 2013 [24]	No direct mobility measures (derived from literature) calculated steps	Not reported	In 10 years, the total medical costs were 5.2 and 8.4% lower for 3,000 and 5,000 steps increase, respectively. The cost reduction associated with a daily increase of 3,000 steps walked was calculated as 0,000,014 $ for each step
Turi et al. 2015 [25]	PRO: Activity of daily living (walking during leisure time as self-reported in retrospective questionnaire ‘Baecke Questionnaire’)	Not reported	Participants inserted in the category of higher involvement in walking were 41% less likely to be inserted into the group with higher total expenditure (r = 0.59; 95% CI 0.39–0.89)
Kabiri et al. 2018 [26]	Step Count No direct mobility measure (derived from MARCHE trial)	The model predicted that a 554-step-per-day increase in mobility would reduce nursing home utilization by 2.8%	The model predicted an increase of 554-step per day increase would reduce total medical expenditures by 0.9%
Karl et al. 2018 [27]	DMO: Step Count (Self-reported hours of exercise per week. Accelerometer (worn during waking hours for 7 days) for uniaxial counts (counts/min) on the vertical axis for the deduction of activity levels and step counts)	Not reported	No significant results (Inactive participants (less than 10,000 steps per day), had higher direct healthcare costs as compared to active subjects)
Bonnini et al. 2020 [28]	PerfO: Walking speed (maintained during a moderate 1-km treadmill-walking test)	Every 1 km/hour increase in walking speed was associated with a 21% reduction in risk of hospitalization (HR 0.79)	Hospitalization costs in the first, second and third propensity score tertile per patient were reduced from 1,281 to 341 $, from 904 to 383 $, and from 1,197 to 334 $ among low and high improvers in walking speed
Okayama et al. 2021 [29]	PerfO: Walking speed (distance covered by walking up and down a 10 m course at a different dictated velocities, termed “incremental shuttle walking distance” (ISWD))	The mobile group had shorter cumulative lengths of hospital stay (41.3 vs. 72.9 days/person) than the less mobile group	The mobile group had lower inpatient medical costs (16,680 $ vs. 25,458 $ /person) than the less mobile group
Hirai et al. 2021 [30]	PRO: Walking Time (categorized average minutes of walking per week by questionnaire)	Not reported	Cumulative LTCI costs were USD 3200 for those who walked for less than 30 min, USD 2400 for those who walked for 30 to 60 min, and USD 2100 for those who walked for more than 60 min

Abbreviations: *PRO* patient reported outcome, *PerfO* performance outcome, *DMO* digital mobility outcome

### Methods used to assess walking ability

The majority of walking assessments consisted of patient-reported outcomes (PROs) collected via questionnaires. These assessments included the following parameters: walking time [21, 22], and walking during leisure time as part of activities of daily life [25]. Specifically, Perkins et al. examined walking time by documenting the minutes of walking per week, using a newly developed questionnaire [21]. Tsuji et al. obtained mobility data from a survey conducted in 1994, which included a question on walking time asking how long on average patients walk a day [22]. Hirai et al. assessed walking time per day with a single question (“How long do you walk a day, on average?”). The time spent walking was categorized as > 60 min, 30–60 min, and less than 30 min per day. Turi et al. assessed walking during leisure time by using the section ‘physical activity during leisure-time’ of the ‘Baecke12’ questionnaire [25]. Walking ability was also assessed by determining walking speed using performance tests. Purser et al. used the Reubens Physical Performance Test, a supervised performance test [23]. Walking speed was examined by Bonnini et al. using a 1-km treadmill-walking test [28], and by Okayama et al. using the Endurance shuttle walk test [29]. Simulation studies did not assess walking directly. They furthermore examined quantitative risk reduction by walking derived from published studies to calculate the steps taken in their cost simulations [24, 26]. The study by Karl et al. measured the number of steps with a portable accelerometer device (Actigraph GT3X) [27].

### Association with health care costs

#### Walking time and leisure-time walking

In a cohort of community-dwelling adults older than 55 years, Perkins et al. found an association between self-reported walking time of 120 min a week or more, and a significant decrease in emergency room visits and hospital stays in the following year. Annual total ($1,856 vs $6,266 $), inpatient ( $1,184 vs $4,872), and emergency room costs ($253 vs $762) were less for those reporting 60 or more minutes of walking per week compared to those reporting less than 60 min of walking per week [21]. In a four-year-long prospective cohort study in Japanese men and women, aged 40–79 years, Tsuji et al. found that medical costs ($86 vs. $97) were 12% significantly lower per capita and month, for subjects walking for more than one hour/day than for those walking less than one hour/day [22]. Hirai et al. reported that time spent walking was negatively associated with the cumulative costs of long-term care insurance. These cumulative costs were significantly higher in those who walked for less than 30 min than in those who walked for more than 60 min. Turi et al. reported the association of self-reported walking during leisure time with total healthcare expenditure during one year prior to the date of the interview in Brazilian patients (randomly selected users of the Brazilian National Health System) aged ≥ 50 years. Individuals who ‘always walked’ were 41% less likely to be in the highest 25% quantile (an indicator of high expenditure) of incurred health-care cost when compared to individuals who ‘never walked ‘ [25] (Table 2).

#### Walking speed

In a frail population of hospitalised medical or surgical patients older than 65 years, Purser et al. found that when the baseline walking speed was 0.10 m/s higher, this was associated with $1,334 lower 1-year costs during the index hospitalization [23]. Bonnini et al. conducted an intervention study to evaluate the effects of an unsupervised home program in patients with cardiovascular disease, consisting of 30–60 min of brisk walking at least 3–4 days per week over 3 years, on rates of hospitalization. Between four and six years after baseline, a significant lower hospitalization rate was observed in patients that had highly improved their walking speed compared to those who had only improved their walking speed to a low extent. This resulted in an average cost reduction per patient between high and low improvers in walking between $489 and $882 [28]. Okayama et al. prospectively enrolled patients aged ≥ 70 years with advanced non-small-cell lung cancer to investigate the association of pre-treatment walking capacity with hospitalization rates and medical costs. During the first year of initial therapy, medical costs (the actual revenue the hospital was paid from the health insurance funds) did not differ between less and more mobile groups, but significantly higher additional inpatients costs ($8,076 per person) were reported for the less mobile group [29].

#### Number of steps

Karl et al. investigated direct medical costs of patients aged between 48 to 68 years. They used cross-sectional data of the population in the German KORA FF4 study. In a subsample of patients for whom daily step count was reported there was no statistically significant difference in costs between those who walked more than 10,000 steps per day and those who did not [27]. Kato et al. 2013 used a Markov model to simulate costs over 10 years for middle-aged Japanese patients with diabetes. They estimated that total medical costs could be 5.2% and 8.4% lower for daily step count increases of 3,000 and 5,000, respectively [24]. Kabiri et al. conducted a microsimulation study of patients aged ≥ 51 years with osteoarthritis, and reported that 554 steps more per day would be associated with a 0.9% reduction in total medical expenditure [26].

## Discussion

Loss of walking-related mobility has an impact on the risk of mortality (most recently Maurice et al. [31]), morbidity, and quality of life, especially in older people, and the association of mobility with readmissions and falls is well reported [3–5, 32]. The costs of impaired walking ability is a major burden for national health systems. Despite this, the observation from Macera nearly 20 years ago, that ‘quantitative data to make the case that medical care costs are lower among individuals who walk than those who do not is scarce’ [33], still holds in 2022.

In this systematic review, we identified ten studies, most of them recent, that reported a relationship between walking ability and health care costs. With one exception, all studies indicated that better walking performance is associated with significantly lower health care costs. Although it is difficult to derive causal relationships from retrospective studies, the results show that maintaining walking ability can be economically beneficial. It also implies that the accurate measurement and assessment of walking ability could be important. In this regard, this review showed clearly that the calculation and reporting of costs varied considerably between studies. Greater standardisation of cost outcomes would have been needed to allow for a comparison of results in a quantitative synthesis (meta-analysis). In addition, methodically different approaches were used to associate walking ability with monetary values. Further heterogeneity was introduced by the use of different sources of cost data: there was a mix of information from institutional electronic health records, insurance databases, direct questionnaires, and other sources.

In our analysis, we focused on quantitative walking measures, e.g. walking speed, walking distance or the number of steps to enable consistency in decisions regarding the inclusion of studies. Yet, the quantification of walking performance using retrospective questionnaires, as employed in a sizeable part of the included studies, suffers from recall bias and may impact the reported associations with health care costs. For some time now, walking outcomes can be recorded by devices such as mobile phones and wristbands or watches, but in this review, we could include only one study which used such a digital mobility outcome [27]. DMOs can record how a patient functions in the real world, and thus cover a meaningful aspect of health. Assuming that they can be shown to meet necessary requirements in terms of reliability, consistency, sensitivity to change and accuracy [34], they are increasingly recognized as having the potential to contribute important endpoints in clinical trials [14].

Not only clinical but also health economic studies would benefit from the availability of DMOs. Due to their high reliability, accuracy, and simple acquisition, DMOs could be used as a tool for HTA and economic evaluation where cost models could relate a change in the walking ability (e. g. walking speed or number of steps taken) to a monetary value. HTA reports could include the value of the expected cost reduction through improved mobility in cost-effectiveness or cost-utility analyses of new interventions. In such analyses, the willingness to pay threshold is used as an economic decision criterion but the ratios set are often not based on scientific evidence [35]. Future research approaches could address the question of the individual or societal willingness to pay for maintaining certain walking-related mobility levels. Some existing limitations of cost–benefit analysis could be addressed with precise and validated correlations of monetary values and health outcomes such as walking performance and clinically predicted manifestations. Taken together, the use of the association of costs and walking outcomes could become more relevant for health policy research, HTA, budget impact calculations for health insurance companies and the negotiation of reimbursement prices. DMOs may also be of value in the context of generating real-world evidence for monitoring actual costs of therapy.

Some limitations to our review apply. Our systemic literature search includes the most relevant databases (PUBMED and EMBASE) for primary research publications in the field of health economic studies, but did not include reviews by HTA bodies or governmental reports. A search in NHSEED and DARE database was not included as these have been discontinued. The search strategy used the main keywords “mobility”, “walking speed”, and “cost” without restriction, while including additional search terms with a title search only. We acknowledge that publications might have been missed that did not include either of those elements in the title. Yet, the framework of the CHEERS checklist clearly recommends that cost analyses should already be identifiable as such in the title. It should also be mentioned that several studies could not be included in the results although they reported cost effects related to general physical activity. In these cases, it was not possible to explicitly extract walking-related mobility outcomes. Moreover, the reported cost effects are based on direct health care costs, originating from different sectors and payers. This means that the studies are very heterogeneous and our review provides no information about the relationship of walking ability to costs beyond this perspective (e.g., indirect costs due to the loss of productivity). Although cost values were converted to USD and inflated to 2021 equivalent values, data were collected in different countries, at various points in time, and against a background of different health care settings. The presence/absence of chronic conditions varied between studies and most of the studies included enrolled middle-aged to older populations. As a consequence, this review cannot inform about the association of walking ability and health care cost in young individuals.

## Conclusion

Our systematic review demonstrates the specific relevance of walking-related mobility for health care costs. Regardless of the type of assessment of walking ability (walking time, leisure-time walking, walking speed, number of steps), the studies show that better walking ability was significantly associated with lower health care costs. Future health economic research and health technology assessments should use quantifiable mobility outcomes when evaluating new drugs or non-pharmacological interventions. Walking parameters such as number of steps or walking speed appear to be particularly well suited for use in economic cost evaluations due to their scalability. They might help to quantify the monetary value of a new therapy in terms of improvement in walking performance as an additional source of information. Further studies are needed, starting with a clear prospective definition of the mobility outcome and greater standardisation of the costing perspective, study design, and analyses. In the future, digital mobility outcomes can provide objective measures of walking ability to inform health technology assessments and payer’s decisions more reliably. 

## Figures and Tables

**Table 6:**
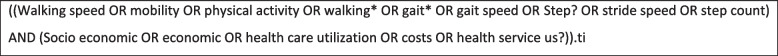
Embase search term

## Data Availability

For all inquiries regarding data access, please contact the corresponding author.

## Appendix

Tables 4, 5 and 6.

**Table 4:** PICO scheme

Population	Studies of participants with no restriction to specific indications, ages, or gender
Comparison	Studies that include health care utilization and/or healthcare costs of patients comparing between patients with different levels of mobility outcome parameters
Outcome	Healthcare utilization and/or health care costs
Region	Any
Language of publication	Abstract available in English
	Full-text available in English or German
Type of study	Health economic studies
Time frame	No restriction

**Table 5:** Medline search terms

Concept	Terms	Hits
Walking Outcomes / Mobility	Walking speed [Mesh] OR mobility [Mesh] OR physical activity [ti] OR walking* [ti] OR gait* [ti] OR gait speed [ti] OR Step? [ti] OR stride speed [ti]	167,436
Economics	Socio economic [ti] OR economic [ti] OR health care [ti] OR costs OR health service use [ti]	900,150
Connection	# AND	3,409
	# NOT editorial OR letter OR historical article OR review.pt	2,216
